# How Confidence in Prior Attitudes, Social Tag Popularity, and Source Credibility Shape Confirmation Bias Toward Antidepressants and Psychotherapy in a Representative German Sample: Randomized Controlled Web-Based Study

**DOI:** 10.2196/11081

**Published:** 2019-04-23

**Authors:** Stefan Schweiger, Ulrike Cress

**Affiliations:** 1 Knowledge Construction Lab Knowledge Media Research Center Leibniz-Institut für Wissensmedien Tuebingen Germany

**Keywords:** attitude, psychotherapy, antidepressive agents, culture, Germany, health literacy, professional competence, information systems, consumer health information, information dissemination

## Abstract

**Background:**

In health-related, Web-based information search, people should select information in line with expert (vs nonexpert) information, independent of their prior attitudes and consequent confirmation bias.

**Objective:**

This study aimed to investigate confirmation bias in mental health–related information search, particularly (1) if high confidence worsens confirmation bias, (2) if social tags eliminate the influence of prior attitudes, and (3) if people successfully distinguish high and low source credibility.

**Methods:**

In total, 520 participants of a representative sample of the German Web-based population were recruited via a panel company. Among them, 48.1% (250/520) participants completed the fully automated study. Participants provided *prior attitudes* about antidepressants and psychotherapy. We manipulated (1) *confidence* in prior attitudes when participants searched for blog posts about the treatment of depression, (2) *tag popularity* —either psychotherapy or antidepressant tags were more popular, and (3) *source credibility* with banners indicating high or low expertise of the tagging community. We measured *tag* and *blog post* selection, and *treatment* *efficacy ratings* after navigation.

**Results:**

Tag popularity predicted the proportion of selected antidepressant tags (beta=.44, SE 0.11; *P*<.001) and blog posts (beta=.46, SE 0.11; *P*<.001). When confidence was low (−1 SD), participants selected more blog posts consistent with prior attitudes (beta=−.26, SE 0.05; *P*<.001). Moreover, when confidence was low (−1 SD) and source credibility was high (+1 SD), the efficacy ratings of attitude-consistent treatments increased (beta=.34, SE 0.13; *P*=.01).

**Conclusions:**

We found correlational support for defense motivation account underlying confirmation bias in the mental health–related search context. That is, participants tended to select information that supported their prior attitudes, which is not in line with the current scientific evidence. Implications for presenting persuasive Web-based information are also discussed.

**Trial Registration:**

ClinicalTrials.gov NCT03899168; https://clinicaltrials.gov/ct2/show/NCT03899168 (Archived by WebCite at http://www.webcitation.org/77Nyot3Do)

## Introduction

### Background

Do people attend to information independent of their prior attitudes, and do they distinguish expert from nonexpert sources on the Web? To address these important questions [1-3], we investigate confirmation bias, the tendency to favorably select and evaluate attitude-consistent information [3-6].

A comprehensive meta-analysis identified 2 major motivational factors that moderate confirmation bias [7]. First, when we face information that suggests our point of view is wrong, we try to maintain our prior attitudes by choosing and believing in attitude-consistent information, which is called *defense motivation* [7-9].

In contrast to this, in some situations, we may be genuinely interested in acquiring objectively correct and accurate information [7,8,10]. This *accuracy motivation* can guide our information search, even when information is not consistent with our prior attitudes [7]. Particularly in the health context, we should form attitudes independent of our defense mechanisms and base evaluations on objectively correct information. In the following sections, we outline 3 factors that may reduce confirmation bias, given that we are accuracy motivated when searching for mental health–related information.

### Confidence and Confirmation Bias

First, low confidence should decrease confirmation bias [7]. However, people tend to be overly confident in prior attitudes and knowledge [11,12] in a large range of domains, such as academic, intellectual, vocational, athletic, and medicine [13]. When people are overly confident in their prior attitudes, confirmation bias increases [14].

For the mental health–related context, it is important that confidence varies for people with different mental disorders [15]. For example, individuals who experience anxious and depressive symptoms show less than average confidence (but average accuracy) in decision-making tasks [15], which suggests that they could be even less prone to confirmation bias.

Looking at how to influence confidence, overconfidence can be reduced when participants reflect on their ability to describe, in a step-by-step manner, the causal functioning of objects to experts [16]. We draw on a manipulation that focused on people recalling situations where they were either confident or doubtful about their own thoughts—study 3 [17]. When participants recalled situations in which they had been confident (vs doubtful), and subsequently provided arguments about a controversial topic, they were more (or less) confident about their arguments [17,18]. For this study, one main goal was to replicate the manipulation (study 3 in [17]) with a representative sample, in the mental health context.

A recent review has shown that confidence manipulations tend to increase confirmation bias, which is explained by the defense motivation account [7]. According to defense motivation, when people have low confidence, they aim to defend their self-concept by selecting information that is in line with their attitudes. In contrast to this, we draw on a metacognitive manipulation of confidence that aims to make people perceive their current thoughts as less valid—study 3 in [17]. Consequently, they should perceive their attitudes as less valid (independent of their self-concept), and confirmation bias should decrease, given that searchers aim for valid information.

We expect that when prior attitudes are held with high confidence, participants preferably select and evaluate attitude-consistent information. If participants were defense motivated, high (vs low) confidence would make them less (vs more) threatened by attitude-inconsistent information and they would select more attitude-inconsistent information and evaluate it more favorably [7].

### Social Tags as Signposts to Popular Information

**Figure 1:**
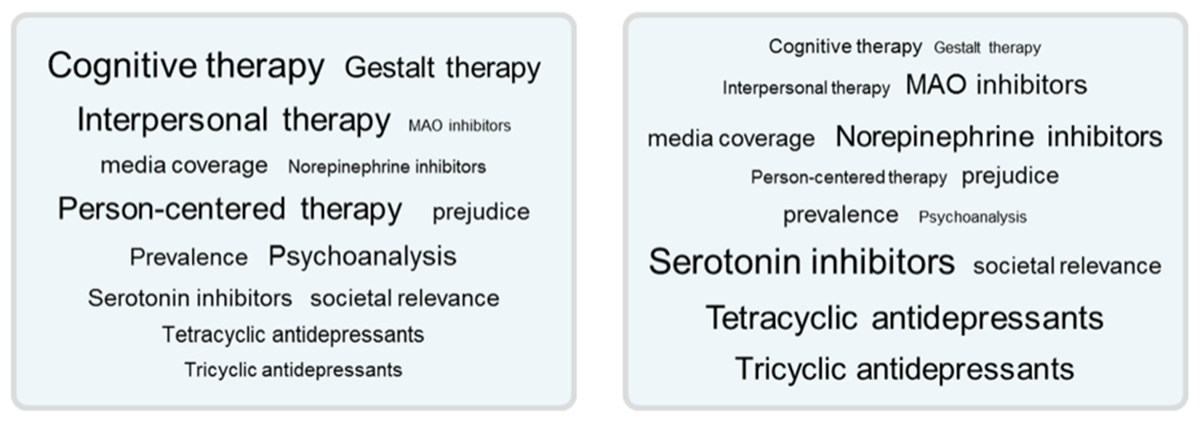
The tag clouds used in the present study. Either psychotherapy (left), or antidepressants (right) were more popular.

The second influence on confirmation bias occurs when people face cues from socially aggregated information on the Web [19-24]. Cues indicating socially aggregated information include star ratings, likes, retweet counts, or social tags. In the case of tagging, tag clouds arise when users label or tag content on the Web, such as videos, images, or documents (Figure 1) [25,26]. When tags from the tagging community are aggregated and presented in tag clouds, the tags represent the consent of a majority of people and guide information searchers [19,20]. High majority consent or high tag popularity translates into large tags, which attract more attention than smaller tags with less social consent.

We suggest that social tag clouds are particularly nonintrusive and therefore highly suited to circumvent the influence of prior attitudes as larger tags are visually dominating, and it has been shown that people who primarily attend to large tags [25,27,28] are more likely to click on large tags [20,29,30] even when large tags are inconsistent with activated associations in memory [29,30] or prior attitudes [20]. Moreover, social consent elicits the behavior that conforms to the majority in offline settings [31,32].

Moreover, people select more trustworthy results when facing a grid-like (vs list-like) arrangement of search results, similar to social tag clouds [33]. In sum, tag clouds should be suited to decrease the influence of prior attitudes in information search and reduce confirmation bias.

### Source Credibility of the Tagging Community

People do not always successfully consider high-quality information [34,35], particularly when browsing user-generated content [34]. A total of 2 meta-analyses concluded that personal characteristics [35], as well as platform characteristics [34], play an important role. The relationship between manipulated source credibility and perceived information credibility is higher for student samples (vs nonstudent samples) [35], and for user-generated content that is presented on common websites (vs blogs and discussion boards) [34].

Besides education, epistemic beliefs can influence how people perceive source credibility. For example, when searching information on 2 competing therapies for Bechterew disease, not all participants took source credibility into account [36]. Particularly, participants who viewed the Web as a reliable (vs unreliable) source of accurate knowledge did not reflect upon source credibility, they viewed URLs for a shorter time and selected less search results at the bottom of the page.

To our knowledge, there is a single study that uses tag clouds to investigate source credibility in the health context [37]. People searched for information on how to manage diabetes on a health forum with tag clouds [37]. In the first task, they searched for information that was of general interest, and in a second task, they searched for credible information. The tag cloud comprised 12 filler tags (eg, glucose, diet, and exercise), and 6 tags indicating source credibility of content (author, date, quote, reference, statistics, and testimonial). When participants browsed for general interest, only one-third used at least a source credibility tag. When explicitly asked to take source credibility into account, 90% used at least 1 source credibility tag.

It remains an open question whether people in a representative sample take the source credibility on a social tagging platform into account. In line with the accuracy motivation account, we expect that if information searchers recognize high source credibility, they will select more tags and related blog posts in total, regardless of whether attitude-consistent or attitude-inconsistent tags are more popular in the social tag cloud. If, on the other hand, people showed defense motivation [7], they would avoid attitude-inconsistent tags and blog posts with high source credibility and evaluate it less favorably.

### Prior Attitudes Toward Antidepressants and Psychotherapy

With respect to the treatment of depressive disorders, people clearly favor psychotherapy over antidepressants [38-44]. Attitudes of laypeople manifest in estimated treatment efficacy as well as treatment recommendations [20,41,43,45,46]. People believe antidepressants to be little to moderately effective, whereas psychotherapy is believed to be moderate to highly effective [20]. As literature shows about equal, moderate efficacy of both types of treatment [47-49], people’s attitudes and recommendations are biased.

We expect more positive prior attitudes toward psychotherapy than toward antidepressants in the German population, and with this study, we aim to describe the magnitude of the psychotherapy preference and present the arguments that shape these biased attitudes.

### Hypotheses

First, we expect that people’s attitudes (H1a) and efficacy ratings (H1b) before navigation are more favorable for psychotherapy than for antidepressants.

We expect to replicate Study 3 in [17]: after recalling situations in which participants were confident (vs doubtful), they should be more confident in their own arguments (H2a). We expect that high (vs low) *confidence* leads to a more pronounced confirmation bias and an increased selection of attitude-consistent tags (H2b) and blog posts (H2c), and this will strengthen the attitudes people already had before navigation (H2d). So, when prior attitudes favor psychotherapy, and confidence is high, participants prefer psychotherapy tags and blog posts and change their attitudes even more toward psychotherapy. If confidence is low, prior attitudes should not be related to selection of tags and blog posts and attitude change.

*Tag popularity* should circumvent the influence of prior attitudes, so participants select popular tags more frequently than less popular tags (H3a) and blog posts (H3b). Consequently, attitudes change in line with tag popularity (H3c).

Participants distinguish high from low *source credibility* (H4a). When tags and blog posts are collected by experts (vs novices), participants click on more tags (H4b) and blog posts (H4c) overall, independent of their prior attitudes, and people should show more attitude change for both treatments (H4d).

## Methods

### Participants

**Figure 2:**
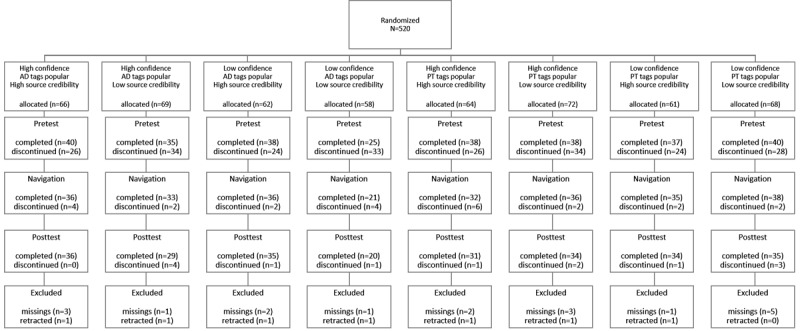
CONSORT flow diagram. AD: antidepressants; PT: psychotherapy.

A representative sample with respect to age and gender was randomly drawn from a pool of a panel company. In total, 520 participants started the fully automated Web-based study, 48.1% (250/520) completed it, 1.3% (7/520) withdrew their data, and 3.2% (17/520) participants were dropped as they did not provide responses (Figure 2). Age of the remaining 43.5% (226/520) participants ranged from 18 to 60 years (mean 40.36, SD 12.17), and 50.0% (113/226) were female (Table 1). With respect to familiarity of the technology used in the study, 24.8% (56/226) stated they were familiar with the term *tag cloud*, 36.7% (83/226) stated they had already clicked on single tags to navigate the Web. Ethical approval was granted by the Ethical Committee of the Knowledge Media Research Center (LEK 2014/006).

**Table 1:** Sample characteristics (N=226).

Characteristic		Statistics, n (%)
**Education**		
	Qualified job	18 (8.0)
	Abitur certificate	102 (45.1)
	University degree	53 (23.5)
	Other	53 (23.5)
**Age in years**		
	18-19	9 (4.0)
	20-29	46 (20.4)
	30-39	45 (19.9)
	40-49	65 (28.8)
	50-59	52 (23.0)
	60	9 (4.0)
**Gender**		
	Male	113 (50.0)
	Female	113 (50.0)

### Procedure and Design

**Figure 3:**
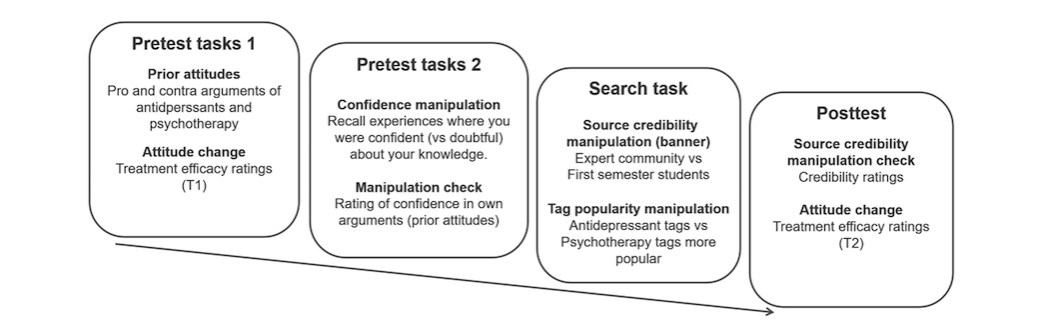
Experimental procedure.

This study comprised a 2 (confidence: high and low) × 2 (tag popularity: antidepressants high and psychotherapy high) × 2 (tagging source credibility: high and low) between-subjects design. Participants enrolled via a Web-based portal of a private panel company (respondi AG, Cologne, Germany; ISO 26362 certified), which linked to our survey, and participants were offered €4 to complete it. First, participants were welcomed and informed that they could withdraw participation at any point. Participants were granted anonymity and asked to provide informed consent by clicking the button to start the study, after which they were randomly assigned to 1 of the 6 experimental conditions by a computerized random number procedure. Then, for *prior attitudes*, we asked participants to state pro and contra arguments regarding antidepressants and psychotherapy (pretest tasks 1; Figure 3). Next, they rated the efficacy of antidepressants and psychotherapy on scales. Then, they provided responses for an allegedly unrelated pilot study, which served to manipulate *confidence* [17]. Participants were asked to recall situations in which they had felt either confident or doubtful about their own knowledge (Study 3 in [17]). After this, they were asked to think back about their arguments regarding psychotherapy and antidepressants and they rated how confident they were about the arguments they had provided before. This rating served as a manipulation check for confidence. Next, participants searched for information about treatment efficacy to provide treatment advice for a hypothetical, closely-related person. To manipulate *source credibility*, we informed them that the blog post and responding tag had been gathered by a community of either experts in the field, such as experienced psychiatrists and by psychotherapists (high source credibility condition), or by psychology students and medical students in their first semester (low source credibility condition). To manipulate *tag popularity*, either psychotherapy or antidepressant tags were larger (Figure 1). They could also provide tags for blog posts. After 5 min of browsing in the tagging environment, a *Next* button appeared and from then on, participants could decide when to stop browsing tags and related blog posts. After navigation, participants rated source credibility (manipulation check) and provided efficacy ratings again. At the end of the study, participants could provide feedback in a text box.

### Materials

For the information search task, we provided a tagging environment (Multimedia Appendix 1). We presented 14 tags on the right side of the screen in which 5 tags represented psychotherapy, 5 tags represented antidepressant treatments, and 4 filler tags were irrelevant for treatment (prejudice, media coverage, societal relevance, and prevalence). Depending on the experimental condition, either psychotherapy-related tags or antidepressants-related tags were larger (ie, more popular). When participants clicked on a tag, 3 blog posts were presented on the left (Multimedia Appendix 1). Each blog post described a symptom of depressive disorders and the scientific studies on the efficacy of the treatment. In a pilot study, we had assured that the blog posts had equal persuasiveness. First, only the headline and the first sentence of each of the 3 related blog posts were shown. To read the full post, participants clicked on *(more...)*.

### Independent Variables

#### Prior Attitudes

As index of pro and contra arguments for psychotherapy and antidepressants, we subtracted the number of arguments favoring antidepressants (contrapsychotherapy and proantidepressants) from the number of arguments favoring psychotherapy (propsychotherapy and contraantidepressants). Positive values of this index thus indicate a preference for psychotherapy. Arguments were rated by 2 raters (*r*=.78; *P*<.001), where differences were resolved by agreement.

#### Confidence

We adapted the experimental procedure by Petty and colleagues (Study 3 in [17]) and participants recalled situations in which they had felt confident or doubtful about their own knowledge, using 5 input text boxes for 5 min.

#### Tag Popularity

For the psychotherapy popular group, psychotherapy tags were larger, and for the antidepressant popular group, antidepressants tags were larger (Figure 1).

#### Source Credibility

**Figure 4:**
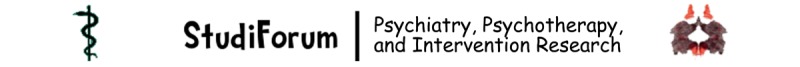
Banner for the low source credibility condition.

**Figure 5:**
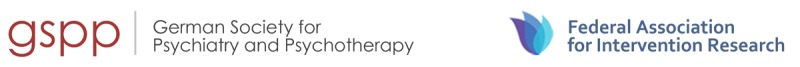
Banner for the high source credibility condition.

On top of the page, banners showed that either alleged college students (low source credibility; Figure 4) or domain experts (high source credibility; Figure 5) had collected and tagged the blog posts. After the search task, participants rated the source credibility of the information on a scale from 1 (not at all) to 7 (highly).

#### Confidence Ratings (Manipulation Check)

After participants listed situations in which they had been confident or unconfident, they rated confidence in their own arguments regarding prior attitudes on a scale from 1 (not at all) to 7 (highly). They were asked how the following words described their arguments: obvious, dubious, justified, credible, factual, well-founded, persuasive, and objective (Cronbach alpha=.88).

#### Source Credibility Ratings (Manipulation Check)

Participants rated the degree to which the following words described the tagging community: informed and competent (*r*=.70; *P*<.001).

### Dependent Variables

#### Efficacy Ratings (Attitude Change)

Participants agreed to statements on the efficacy of psychotherapy and antidepressants on a scale from 1 (completely disagree) to 7 (completely agree), before (antidepressants Cronbach alpha=.89 and psychotherapy Cronbach alpha=.92) and after navigation (antidepressants Cronbach alpha=.94 and psychotherapy Cronbach alpha=.95). To predict attitude change with respect to treatment preference, we derived a difference index score, subtracting the antidepressant from psychotherapy treatment ratings.

Beside attitude change in terms of treatment preference, we analyzed pooled attitude change by taking the sum of efficacy ratings for both treatments before and after navigation (divided it by the number of items for interpretability).

#### Tag and Blog Post Selection

To measure attitude-consistent navigation, we recorded the number of tags and blog posts selected for each treatment category (0=psychotherapy and 1=antidepressants).

## Results

All analyses presented were conducted with the R Software (R Foundation for Statistical Computing; Version 3.3.4); raw data and the analysis script can be found in Multimedia Appendix 2.

### Prior Attitudes

**Figure 6:**
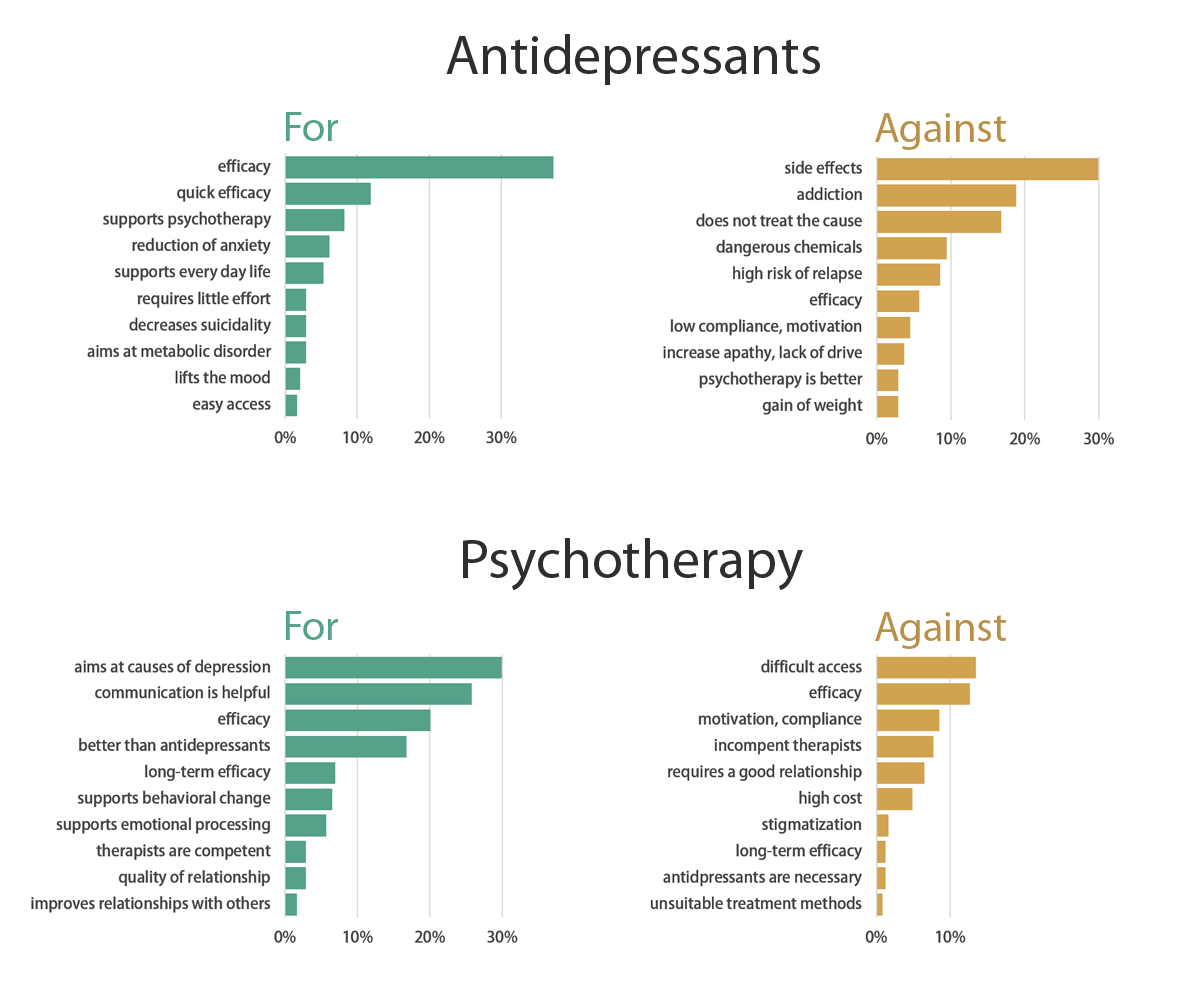
Arguments for and against the 2 treatments.

As expected in H1a, we found that participants’ prior attitudes favor psychotherapy over antidepressants. Participants stated more arguments for psychotherapy (mean 1.69, SD 1.77) than for antidepressants (mean 1.06, SD 1.51; *t*_225_=5.30; *P*<.001, *d*=0.26), and they stated more arguments against antidepressants (mean 1.51, SD 1.53) than against psychotherapy (mean 0.73, SD 1.54; *t*_225_=8.13; *P*<.001, *d*=0.34). We also descriptively analyzed arguments and pooled them into qualitative categories (Figure 6).

**Figure 7:**
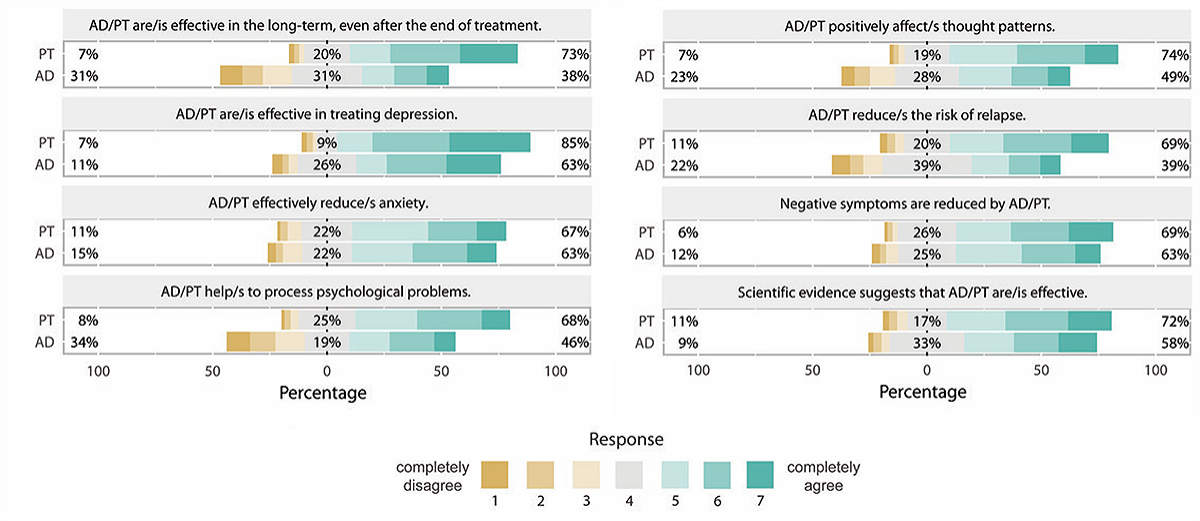
Prior attitudes about psychotherapy and antidepressants before information search. AD: antidepressants; PT: psychotherapy.

With H1b, we expected that people would provide more favorable efficacy ratings for psychotherapy compared with antidepressants before navigation. Participants rated statements about the efficacy of both treatments on 8 items, on a scale from 1 to 7 (Figure 7). As the internal consistency was high for both scales (antidepressants Cronbach alpha=.89 and psychotherapy Cronbach alpha=.92), we pooled them. A paired *t* test showed a moderate effect on the preference for psychotherapy (mean 5.24, SD 1.10) over antidepressants (mean 4.61, SD 1.19; *t*_225_=9.71; *P*<.001, *d*=0.56; see items and response distribution in Figure 7). In sum, prior attitudes measured via pro and contra arguments, as well as via efficacy ratings, favored psychotherapy over antidepressants. Both measures were moderately correlated (*r*=.41; *P*<.001).

### Manipulation Checks

#### Confidence

Contrary to our expectations (H2a), we could not replicate the confidence manipulation (Study 3 in [17]). After recalling situations in which they had been confident (mean 4.64, SD 1.20), participants were not more confident about their arguments compared with recalling situations in which they had been doubtful (mean 4.68, SD 1.09; *t*_224_=<1; *P*=.78).

#### Source Credibility

In contrast to our expectation (H4a), source credibility ratings in the high source credibility condition (mean 4.87, SD 1.26) did not significantly differ from source credibility ratings in the low source credibility condition (mean 5.16, SD 1.32; *t*_224_=1.67; *P*=.10).

As the confidence and source credibility manipulations were ineffective, we used respective manipulation check scores in the following regression analyses as predictors.

### Confidence in Prior Attitudes

#### Tag Selection

To analyze attitude-consistent and attitude-inconsistent tag selection, we conducted logistic regressions with the dependent variable clicks on treatment tags. The number of clicks on the respective treatment (0=psychotherapy tag selected and 1=antidepressant tag selected) was entered in a logistic regression (Table 2). As predictors, we entered *prior attitudes* and *tag popularity* (0=psychotherapy tags popular and 1=antidepressant tags popular), *confidence ratings*, and *source credibility ratings* (see independent variables). We included 2-way interaction terms (Step 2 in Table 2) and tested for interactions with likelihood ratio tests [50,51].

**Table 2:** Selection ratio of antidepressant tags.

Predictor	Step 1^a^			Step 2^b^		
	Beta^c^	SE	*P* value	Beta	SE	*P* value
Intercept	−.39	0.08	<.001	−.41	0.08	<.001
Prior attitudes	−.02	0.03	.37	−.03	0.03	.32
Confidence score	.002	0.05	.97	.04	0.05	.82
Tag popularity	.44	0.11	<.001	.44	0.11	<.001
Source credibility score	−.005	0.04	.92	−.005	0.04	.92
PA^d^×confidence score	—^e^	—	—	−.01	0.03	.65

^a^Model fit: χ^2^_4_=17.1; *P*=.002 (Step 1).

^b^Model fit change: χ^2^_1_=0.2; *P*=.65 (vs Step 2).

^c^Continuous predictors were centered.

^d^PA: prior attitudes.

^e^Interaction term not included.

We expected that high confidence should strengthen the relationship between prior attitudes and the proportion of clicks on attitude-consistent tags (H2b). However, there was no significant interaction of the predictors’ confidence in prior attitude ratings and prior attitudes predicting the selection of antidepressant tags (Step 2 in Table 2). As likelihood ratio tests showed, including 3-way interaction (χ²_10_=4.9; *P*=.90) and 4-way interaction (χ²_11_=5.0; *P*=.93) did not improve model fit.

#### Blog Post Selection

**Figure 8:**
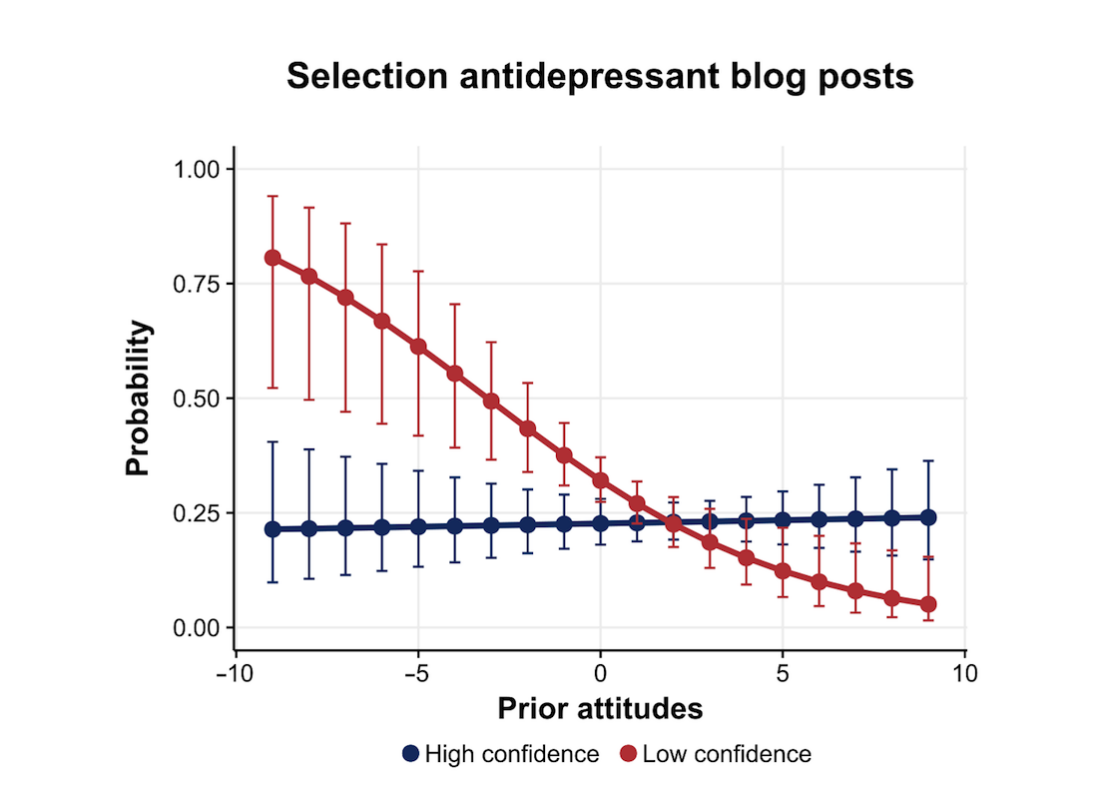
Predicted proportion of antidepressant blog posts selected, for high (+1 SD) and low (−1 SD) confidence (95% CI), with negative values indicating a preference for antidepressants over psychotherapy.

A second logistic regression used the same predictors as in the regression predicting tag selection but with blog post selection as criterion variable (Table 3). We expected that high confidence should strengthen the impact of prior attitudes and consequently lead to increased proportion of clicks on attitude-consistent blog posts (H2c). We observed an interaction between confidence and prior attitudes (beta=.11, SE 0.02; *P*<.001). To disentangle the interaction, we compared slopes of high (+1 SD) and low (−1 SD) confidence ratings. This showed that when confidence ratings were low (−1 SD), participants selected a higher proportion of blog posts that were in line with their prior attitudes (beta=−.26, SE 0.05; *P*<.001; Figure 8). When confidence ratings were high (+1 SD), there was no association with prior attitudes (beta=.02, SE 0.03; *P*=.57; Figure 8). In contrast to our expectation, and in line with the defense motivation account, when confidence was low but not high, there was an association between prior attitudes and selection of attitude-consistent blog posts.

Compared with the model including the 2-way interaction term (Step 2 in Table 3), neither including 3-way interaction term (χ²_4_=5.8; *P*=.21) nor including the 4-way interaction term (χ²_5_=6.0; *P*=.31) yielded a better model fit (all respective lower-order interaction terms were included as well).

**Table 3:** Selection ratio of antidepressant blog posts.

Predictor	Step 1^a^			Step 2^b^		
	Beta^c^	SE	*P*	Beta	SE	*P*
Intercept	−.75	0.07	<.001	−.87	0.08	<.001
Prior attitudes	−.05	0.03	.06	−.12	0.03	<.001
Confidence score	−.11	0.05	.02	−.04	0.05	.46
Tag popularity	.44	0.11	<.001	.45	0.11	<.001
Source credibility score	.02	0.04	.73	.03	0.04	.52
PA^d^×confidence score	—^e^	—	—	.11	0.02	<.001

^a^Model fit: χ^2^_4_=30.4; *P*<.001.

^b^Model fit change (vs Step 1): χ^2^_1_=25.6; *P*<.001.

^c^Continuous predictors were centered.

^d^PA: prior attitudes.

^e^Interaction term not included.

#### Attitude Change

We conducted multiple linear regressions. First, with the predictor variables prior attitudes, confidence ratings, and source credibility ratings (all centered), and the dichotomous variable tag popularity (0=psychotherapy popular and 1=antidepressants popular). In addition, we included the predictor difference score of efficacy ratings (antidepressants subtracted from psychotherapy) before navigation to analyze attitude change with a covariate approach [52]. As a criterion for attitudes after navigation, we included the difference score of efficacy ratings (Table 4).

**Table:** Treatment efficacy ratings (psychotherapy-antidepressants) after navigation.

Predictor	Step 1^a^			Step 2^b^			Step 3^c^		
	Beta^d^	SE	*P* value	Beta	SE	*P* value	Beta	SE	*P* value
Intercept	.70	0.08	<.001	.73	0.08	<.001	.74	0.08	<.001
Efficacy ratings (PT^e^−AD^f^) before navigation	.79	0.06	<.001	.78	0.06	<.001	.79	0.06	<.001
Tag popularity	−.05	0.12	.64	−.06	0.12	.60	−.05	0.11	.65
PA^g^	.08	0.03	.01	.10	0.06	.005	.12	0.03	<.001
Confidence score	−.08	0.05	.16	−.10	0.06	.08	−.10	0.06	.07
Source credibility score	.04	0.05	.34	.05	0.05	.33	.11	0.05	.03
PA×confidence score	—^h^	—	—	−.04	0.03	.16	−.07	0.03	.02
PA×source credibility score	—	—	—	—	—	—	.02	0.02	.40
Confidence score×source credibility score	—	—	—	—	—	—	−.03	0.04	.53
PA×confidence score×source credibility score	—	—	—	—	—	—	−.07	0.02	<.001

^a^Model fit: adjusted *R*^2^=0.49, *F*_5,220_=43.68; *P*<.001.

^b^Model fit (vs Step 1): ∆ adjusted *R*^2^<0.01, *F*_1,219_=1.94; *P*=.16.

^c^Model fit (vs Step 2): ∆ adjusted *R*^2^=0.02, *F*_3,216_=3.88; *P*=.01.

^d^Continuous predictors were centered.

^e^AD: antidepressants.

^f^PT: psychotherapy.

^g^PA: prior attitudes.

^h^Not applicable.

**Figure 9:**
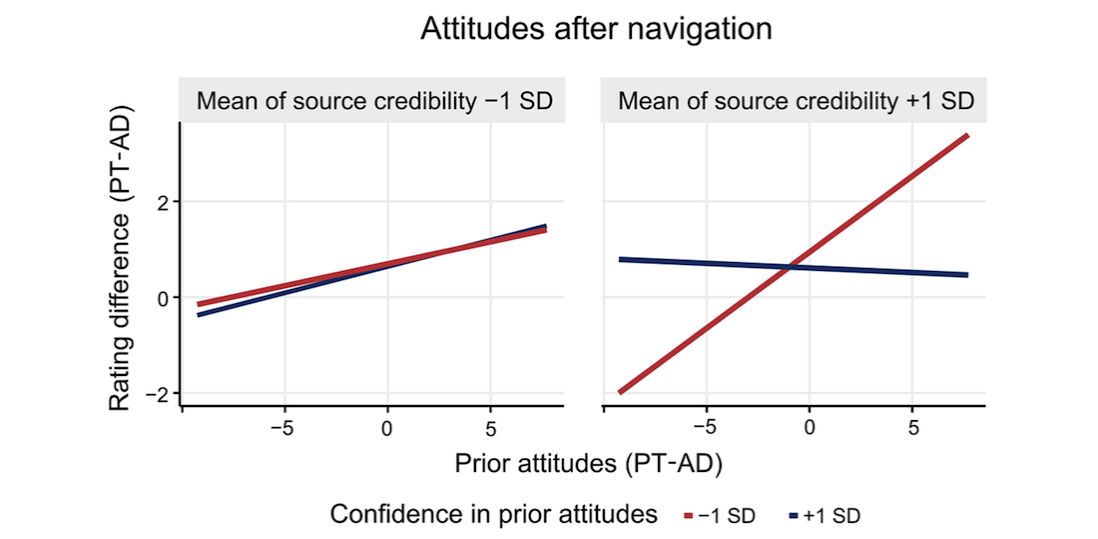
Prior attitudes, confidence and source credibility, and treatment efficacy ratings after navigation, with negative values on all axes indicating a preference for antidepressants over psychotherapy. AD: antidepressants; PT: psychotherapy.

We expected that high (vs low) confidence would lead to higher confirmation bias and decreased attitude change; therefore, for people who hold their attitudes with high (vs low) confidence, prior attitudes should be more strongly associated with attitudes after navigation (H2d). The expected interaction between confidence and prior attitudes was not significant (Step 2 in Table 4). However, the association between confidence and prior attitudes depended on source credibility (Step 3 in Table 4). To disentangle this 3-way interaction, simple slopes were tested on low (−1 SD) and high (+1 SD) levels of source credibility ratings and confidence ratings. This revealed a strong association between prior attitudes and treatment efficacy ratings after navigation for participants with lower ratings of confidence (−1 SD) and high source credibility ratings (+1 SD; beta=.34, SE 0.13; *P*=.01) but no association for high confidence ratings (+1 SD) and low source credibility ratings (−1 SD; beta=.11, SE 0.06; *P*=.053). There was also no association with low confidence (−1 SD) and low source credibility ratings (−1 SD; beta=.08, SE 0.10; *P*=.42) and with high confidence (+1 SD) and high source credibility ratings (+1 SD; beta=−.04, SE 0.06; *P*=.50; Figure 9).

### Tag Popularity of Treatments in the Social Tag Cloud

#### Tag Selection

In H3a, we expected that popular treatment tags would be selected more often, independent of prior attitudes. To test this, we used a logistic regression model as described in the previous confidence section on tag selection (see Table 2, Step 1). Tag popularity was the only significant predictor for the proportion of selected antidepressant tags (beta=.44, SE 0.11; *P*<.001). This supports H3a, as participants selected a larger proportion of popular tags in the tag cloud. They did this independent of their prior attitudes, as prior attitudes were not associated with tag selection.

#### Blog Post Selection

We also expected that participants would select more blog posts when related tags were more popular (H3b). We tested this with the logistic regression as described in the previous confidence section on blog post selection (see Table 3). This showed that participants selected a larger proportion of blog posts when related tags were popular in the tag cloud, supporting H3b (beta=.44, SE 0.11; *P*<.001; Table 3, Step 1).

#### Attitude Change

We expected in H3c that the attitude change would depend on tag popularity. More popular tags for a treatment should be associated with higher ratings of treatment efficacy. We conducted a linear regression analysis, as described in the previous confidence section (also see Table 4). We did not find an influence of tag popularity on efficacy ratings after navigation (beta=−.05, SE 0.12; *P*=.64; see Table 4, Step 1).

### Source Credibility of the Tagging Community

#### Tag Selection

We expected that when the tagging community comprises experts (vs novices), participants click on more tags (H4b). To test this, we conducted a negative binomial regression with the continuous, centered predictors (source credibility score, prior attitudes, and confidence score), the dichotomous predictor (tag popularity; 0=psychotherapy tags popular and 1=antidepressant tags popular), and the dependent variable (number of selected tags; Table 5). We did not find support for H4b, as the total number of selected tags was not associated with source credibility ratings.

**Table 5:** Tags and blog posts selected.

Predictor	Tags selected^a^			Blog posts selected^b^		
	Beta^c^	SE	*P* value	Beta	SE	*P* value
Intercept	1.81	0.07	<.001	1.16	0.47	.01
PA^d^	.01	0.03	.63	.03	0.04	.48
Confidence score	−.02	0.05	.62	.06	0.08	.43
Tag popularity	.14	0.10	.18	−.34	0.18	.06
Source credibility score	.03	0.04	.51	.02	0.07	.79

^a^Model fit: χ^2^_4_=2.7; *P*=.61.

^b^Model fit: χ^2^_6_=5.1; *P*=.28.

^c^Continuous predictors were centered.

^d^PA: prior attitudes.

#### Blog Post Selection

We expected that when the tagging community comprises experts (vs novices), participants click on more blog posts (H4c). We conducted another negative binomial regression with the continuous, centered predictors (source credibility score, prior attitudes, confidence score), the dichotomous predictor (tag popularity: 0=psychotherapy tags popular and 1=antidepressant tags popular), and the dependent variable (total number of selected blog posts; Table 5). As with the number of selected tags, perceived source credibility did not predict the total number of selected blog posts, not supporting H4c.

#### Attitude Change

When the tagging community comprised experts (vs novices), we expected that participants should change their prior attitudes to a greater degree (H4d). We conducted a linear regression analysis with efficacy ratings before navigation (PT−AD) as a covariate [52] and included the predictors tag popularity (0=psychotherapy tags popular and 1=antidepressant tags popular), prior attitudes, confidence, and source credibility ratings. As a criterion, we included the efficacy rating difference (PT−AD) after navigation. The covariate and the continuous predictor variables were centered [53]. High perceived source credibility was associated with higher treatment efficacy ratings after navigation (Table 6), supporting H4d.

**Table 6:** Treatment efficacy ratings (antidepressants+psychotherapy) after navigation.

Predictor	Ratings T2 (AD^a^+PT^b^)^c^		
	Beta^d^	SE	*P* value
Intercept	10.26	0.12	<.001
Efficacy ratings before navigation (AD+PT)	.76	0.05	<.001
Tag popularity	.02	0.18	.93
PA^e^	.08	0.04	.06
Confidence in PA score	.14	0.09	.12
Source credibility score	.24	0.07	<.001

^a^AD: antidepressants.

^b^PT: psychotherapy.

^c^Model fit: adjusted *R*^2^=.16, *F*_5,220_=9.47; *P*<.001.

^d^Continuous predictors and criteria were centered.

^e^PA: prior attitudes.

## Discussion

### Principal Findings

With this randomized, controlled study, we aimed to investigate prior attitudes about antidepressants and psychotherapy and the tendency to confirm prior attitudes when selecting and evaluating mental health–related information. We presented 3 factors to counter confirmation bias: popularity of treatment tags in a social tag cloud, confidence, and the source credibility of the tagging community. We expected that people would select and favorably evaluate attitude-inconsistent content when confidence was low (vs high). In addition, we expected that *source credibility* and *tag popularity* should influence selection of tags independent of prior attitudes. We could not replicate the confidence manipulation (Study 3 in [17]) and participants did not distinguish source credibility as presented by banners; therefore, we used manipulation check scores for correlational analyses.

As expected, people in the German population rated psychotherapy as more effective than antidepressants, and they reported according beliefs. Increasing tag popularity increased selection of tags, independent of prior attitudes and confidence. In contrast to our expectations, higher source credibility was not associated with increased tag or blog post selection. Participants with high confidence were more open to select attitude-inconsistent blog posts, which is in line with the defense motivation account but not with the accuracy motivation account we had expected [7]. Moreover, we found that people with low confidence rated treatment efficacy in accordance with their prior attitudes but only when perceived source credibility was high.

### Social Tags to Reduce Confirmation Bias

We expected that social tag clouds are a nonintrusive interface to circumvent prior attitudes, and popular tags would be selected more often independent of prior attitudes. We found that people selected popular tags and related blog posts more often. We think that these findings highlight the important role of popular content on the Web and also in the context of mental health–related selection of information. When two treatment options are presented to a searcher, searchers will be guided by more popular information, even independent of their prior attitudes. This could help to design Web-based platforms in which it is desirable to minimize the influence of prior attitudes and maximize the influence of a community.

A thorough discussion about nudges is beyond the scope of this paper, but we consider implications of implementing tag clouds as nudges. Though nudges are controversial in general [54], educational nudges aiming to aid people in making better decisions are less controversial [54]. Moreover, in the health context, it is argued that it is impossible not to be influenced by policies of different stakeholders in general [55]. The way in which tools such as tag clouds influence behavior might be considered more controversial as large tags automatically attract the searchers’ attention [25], thus influencing information selection [21], and therefore tags may restrict deliberate individual agency [54,56].

### Defense Motivation in Mental Health–Related Information Search

We expected that people would be guided by accuracy motivation when searching for mental health–related information. People would strive to select and evaluate information that is objectively correct, regardless of their prior attitudes. In contrast to this, the pattern of results suggests that information searchers were defense motivated, and they tended to confirm their prior attitudes to avoid dissonant cognitions and to maintain a positive view of themselves [7,10,57].

This was reflected in blog post selection and resulting attitude change. We found that low confidence was associated with selecting attitude-consistent blog posts, which suggests that participants may have felt increased threat under low confidence.

The findings on attitude change provide further support for the defense motivation account. People with high confidence were expected to change their attitudes in line with their prior attitudes. However, we found the opposite. When confidence was low, not high, people’s attitudes after navigation were polarized in line with their prior attitudes. However, in contrast to blog post selection, this pattern was only found when source credibility was high but not when source credibility was low. This suggests that attitude-inconsistent information could have posed a double threat when source credibility was high, in combination with low confidence. In all other instances, there was no association between prior attitudes and attitude change.

What follows from defensive processing? Not only when information presents a direct threat (eg, antismoking images) but also when different treatment options are available, prior attitudes have an impact on Web-based information search. When information acknowledges prior attitudes of the reader, the need to maintain a positive self-view can be reduced, and the reader becomes more open to attitude-inconsistent information [58,59]. Therefore, content authors could anticipate the attitudes of their readers when providing health information and acknowledge existing attitudes and views before providing potentially conflicting information.

### Source Credibility and Confirmation Bias

People do sometimes recognize source credibility on the Web [33,34,36]; however, participants did not rate practitioners with years of experience as more credible compared with students of health-related subjects in their first semester. One possible explanation for this is that the banners on top of the page were too subtle.

Moreover, for student samples (vs nonstudent samples) [35] and content that is presented on common websites (vs user-generated content) [34], searchers perceive experts as more credible. This might explain that for this representative sample on a specific tagging platform, people did not distinguish high from low source credibility.

For content authors, this finding underlines the importance to consider the target audience as well as the impact of the type of platform that is being used to convey health-related messages. Although information searchers with high educational background or searchers on general websites respond more to expertise, searchers on sites presenting user-generated content (eg, forums or blog posts) respond more to demographic similarity to the searcher [34], and nonstudent searchers respond less to expertise when judging source credibility.

### Attitude Confidence and Confirmation Bias

A recent study showed that individuals with depressive and anxiety symptoms exhibited lower confidence in a decision-making task [15]. In this study, people with lower confidence evaluated information content in line with their prior attitudes, when the source of information was highly credible. Therefore, when searchers particularly perceive information as highly credible, individuals with depressive or anxiety symptoms might be prone to select attitude-consistent information. This should also be tested by future studies.

### Public Attitudes Toward Antidepressants and Psychotherapy

As for student [20] and representative samples in Germany [38], we also expected prior attitudes to be more positive for psychotherapy than for antidepressants, and we found an according moderate effect. The results about the specific beliefs show that people are not satisfied with the current accessibility of mental health care services, and the German population seems to have specific beliefs when it comes to side effects of antidepressants. However, side effects that can be found in the literature, such as nausea, insomnia, somnolence, fatigue, sexual dysfunction, and weight gain [60,61], were rarely associated with antidepressants.

### Limitations

According to the Federal Office of Statistics, the sample from this study is representative for gender and age, but participants with lower education, such as people with a qualified job, are underrepresented, whereas participants with a university degree are slightly overrepresented [62]. Therefore, the results of this study should be interpreted with caution for people with lower-level education. The recruitment process of the panel company uses Web-based campaigns, search engine marketing, and offline recruitment, where participants register at a portal through which they can enroll for studies that match their demographics. Therefore, it should be noted that this sample is restricted to Web-based users of the German population.

This study suggests that the results for confidence and its interplay with source credibility are in line with predictions of defense motivation; however, because of the correlational design, potential correlated confounding influences could be at work and could potentially have been overlooked.

Moreover, all blog posts highlighted the efficacy aspect of prior attitudes, whereas other important issues such as side effects or treatment of psychological causes were not mentioned in the blog posts. Thus, only one aspect related to prior attitudes, namely treatment efficacy, was addressed in the blog posts. In addition, all blog posts were formulated positively, such that information revealing limitations and boundary conditions of the treatments were addressed in the blog posts.

As age could be an important covariate in this study, we exploratively checked the influence of age for each dependent variable; however, age was not a significant predictor in none of the analyses.

### Conclusions

We presented correlational support for the defense motivation account in health-related search. That is, participants tended to confirm their prior attitudes when searching for information. We presented factors that influence this confirmation bias. First, social tags reduced the influence of prior attitudes, and second, attitude confidence increased confirmation bias when source credibility was high. These findings have many implications for content creators, who should acknowledge existing attitudes in persuasive communication and consider demographics of their audience as well as the type of platform where content is published. Future studies should test whether this result extends to other health-related domains, beyond treatment of depression, and to other information platforms as well. Furthermore, it would be highly interesting to compare treatment attitudes toward internet-based psychotherapy including different delivery modes.

## Appendix

Multimedia Appendix 1 Screenshot of the social tagging environment used in the current study.

Multimedia Appendix 2 Original data and R analysis file.

Multimedia Appendix 3 CONSORT-eHEALTH checklist (V 1.6.1).
