# Supplementary material for: How Confidence in Prior Attitudes, Social Tag Popularity, and Source Credibility Shape Confirmation Bias Toward Antidepressants and Psychotherapy in a Representative German Sample: Randomized Controlled Web-Based Study
Source: J Med Internet Res. 2019 Apr 23;21(4):e11081. doi: 10.2196/11081 (PMC6658248; doi:10.2196/11081)
Supplement: Multimedia Appendix 1 [file jmir_v21i4e11081_app1.pdf]

iwm kmrc
Institut für Wissensmedien
Knowledge Media Research Center

Information Search

Next

Transform Positive Attitudes into Behaviour

Cognitive behavior therapy is effective because patients learn to act on their positive attitudes which they gained in therapy. This is the conclusion from a study of the university of Hamburg, in which Data from a large number of scientific studies of the past 10 years were analyzed. "Patients learn to direct their feelings and thoughts towards a more positive direction. Patients can act on their newly acquired attitudes in every day life," explains Dr. Manfred Lang.

My Tag | *Save Tag*

Less Cognitive Distortions

In Cognitive Behavior therapy patients learn to become aware of their distorted thoughts and irrational beliefs (more...)

To overcome obsessive thoughts

"The efficacy of behavior therapy is based on the fact that people learn to overcome their stable, depressive thought patterns," (more)

Cognitive therapy Gestalt therapy
Interpersonal therapy MAO inhibitors
media coverage Norepinephrine inhibitors
Person-centered therapy prejudice
Prevalence Psychoanalysis
Serotonin inhibitors societal relevance
Tetracyclic antidepressants
Tricyclic antidepressants
